# Energy Levels and SpectSSrum of Neutral Helium (^4^He I)

**DOI:** 10.6028/jres.064A.003

**Published:** 1960-02-01

**Authors:** William C. Martin

## Abstract

A table of energy levels based on the most accurate observations now available is given for the neutral ^4^He atom. The wavelengths contained in a revised list of ^4^He I lines from 320 A to 21132 A are also based on the best measurements, over half of them having been calculated from the wavenumber separations of appropriate levels. Several previously disturbing features of the ^4^He term scheme are obviated by the revised level values. In a discussion of the experimental results for this spectrum some comparisons with theory are made.

## 1. Introduction

It seems important that an up-to-date helium line list and a table of revised energy levels be made generally available. The use of helium in many kinds of spectroscopic sources, in studies of electrical discharge mechanisms and of plasma conditions, and the importance of helium in astrophysics combine to make He I one of the spectra most frequently observed. The most recent essentially complete compilation of helium wavelengths, contained in *A Multiplet Table of Astrophysical Interest* and *An Ultraviolet Multiplet Table* [1],^1^ appeared in 1945 and 1950. Although these lists have advantages over earlier compilations [2, 3] in having line classifications in modern notation and containing more recent wavelength values, improved measurements of many He I lines have been made since they were published. The most notable of these are probably Herzberg’s [4] recent observations, which have decreased the uncertainty in the connection between the ground state and the higher levels about one hundredfold. His measurements, combined with others made since the publication of volume 1 of *Atomic Energy Levels* [5], have led to the present complete revision of the He I level values. These new level values allow the calculation of helium wavelengths which, for over half the known lines, are believed to be more accurate than the available observed wavelengths.

In pointing out the need for the present revision of the He I level values, it may be well to emphasize the special place of two-electron atoms in atomic theory. Since these are the simplest atoms for which exact calculations in either nonrelativistic or Dirac's relativistic wave mechanics cannot at present be made, they furnish excellent testing grounds for the various approximation methods of quantum mechanics and theories of the interaction between electrons. Indeed, until the recent beautiful measurements by Herzberg [4] the theoretically predicted ground-state energy of helium was more accurate than the experimental determination. The fact that Herzberg was able to verify field theory (Lamb) effects in two two-electron atoms (^4^He and ^3^He) illustrates the importance of maintaining as accurate data on the helium-type atoms as contemporary experimental techniques allow.

Naturally, the present discussion of experimental results will stress the more recent observations. The references are mainly to work done since the appearance of volume VII of the *Handbuch der Spectroscopie* [3]. Since the article on helium in that superb work contains a practically complete list of references through 1927, it and this paper together furnish a guide to the principal experimental energy level deternations for ^4^He I to date.

No attempt has been made in this paper to deal with the interesting and important ^3^He I spectrum. Because of the large percentage mass difference of ^3^He and ^4^He and because ^3^He has a nonzero nuclear magnetic moment giving rise to hyperfine structure, the ^3^He I spectrum differs in detail a good deal from ^4^He I. A reasonably comprehensive article on the experimental results for ^3^He I would be about as long as this one. Perhaps one of the investigators of that spectrum will supply such an article.

## 2. Spectrum

Except for a few normally very weak “forbidden” lines, table 1 contains wavelengths for all He I lines which have been observed in the laboratory. It has already been mentioned that well over half these wavelengths were calculated from newly-derived energy level values (see the following section). In the first column of the table, opposite each calculated wavelength, appears a symbol denoting the source of what is probably the most accurate measurement of the line. The symbol in the second column gives the actual source of each wavelength in the table. The meanings of the symbols are given in the appendix following the table.

The wavelengths of the vacuum ultraviolet lines were calculated from adopted level values except for the line at 320.392 A. Wu [6] has made a convincing argument for assigning this line to the transition give in the table, Edlén’s [7] suggested classification, 1*s2s* ^3^S—2*s*2*p* ^3^P°, is open to objection since the upper term is subject to autoionization into the ^3^P° continuum above the 1*s*∞*p* series limit. A weaker line observed by Kruger [8] at 357.507 A has been the subject of speculation by Kruger himself and by Wu [6] and Holøien [9]. These last two authors find that none of the suggested transitions satisfactorily accounts for both the position and width of the line. For this reason it is not listed here. (Both Wu and Holøien incorrectly state that Compton and Boyce observed this line.)

The original investigation of the helium spectrum in the vacuum ultraviolet was made by Lyman [11], who discovered the resonance series and the intercombination line 591 A. Suga [12] has obtained the resonance series 1*s*^2 1^S_0_*—np* ^1^P°_1_ to *n*=15, as well as the forbidden transitions 1*s*^2 1^*S*_0_*—ns* ^1^*S*_0_ to *n* = 7, 1*s*^2 1^S_0_—2*p*, 3*p* ^3^P°_1_, and 1*s*^2 1^*S*_0_—*3d* ^1^D_2_. Bomke [13] had earlier found the lower members of these forbidden series. The most accurate of the older measurements of the He I spectrum in the vacuum ultraviolet were those of Hopfield [14], of Kruger (as given by Paschen [15]), and of Boyce (measurements of the lines 584 A and 537 A given by Boyce and Robinson [16]). Paschen calculated the best absolute value for 1*s*^2 1^*S*_0_ as 198307.9 cm^−1^ from Kruger’s measurements. From this adopted value for the ground term he then calculated wavelengths for the 1*s*^2 1^*S*_0_—*np*^1^P°_1_ series to *n=2.* Using this procedure, which is essentially that followed here, Boyce and Robinson [16] obtained from Boyce’s measurements of the lines 584 A and 537 A what were undoubtedly the best values for the higher resonance lines previous to this paper. They are systematically below the wavelengths in table 1 by only 0.004 to 0.005 A.

The earlier wavelength measurements made in the vacuum ultraviolet region are, of course, superseded by the work of Herzberg [4] and by the values in table 1 based on his work. Herzberg conservatively estimated the uncertainty in his measurements of the lines at 584, 591, and 537 A to be 0.0005 A.

The intensities for the vacuum ultraviolet lines in table 1 are based on those given by Hopfield [14], Compton and Boyce [10], and Kruger [8]. The spectrograms published by Suga [12] were used in an attempt to put these values on a consistent scale. It should be remembered that the relative intensities of these lines are very strongly dependent on the excitation conditions. For most sources, the decreases in the intensities of the higher series members relative to the lowest member would be much sharper than is indicated in table 1. The intensities and, indeed, the appearance or nonappearance of the forbidden lines are especially affected by source conditions.

Care has been taken to assure that the wavelengths given for lines in the air region should also be the best values now available. Almost all of the 192 lines given for this region have been observed, but wavelengths calculated from adopted level differences are considered more accurate than observed values for about half of these. Most of the wavelengths which are taken directly from observation also correspond exactly to the level differences involved, since they were used to determine these differences. Besides the 100 calculated air wavelengths, the table includes 27 other new air values; these are mostly either unpublished measurements by Shenstone [17] or weighted averages from the literature. The wavenumbers given for some of the calculated lines and for several lines which are averages from observations do not agree in the last significant figure with the wavenumbers which would be obtained by applying the Edlén conversion formula to the corresponding wavelengths in table 1. In these cases it is the *wavenumber* which is the primary adopted quantity, and the wavelength is taken from the Edlén formula to the number of figures regarded as significant.

Only two “forbidden” lines in the air region are- given in table 1. These are measurements by Herzberg [4] of the intercombinations used to determine the relative positions of the singlet and triplet systems. In addition to other intercombination lines, numerous transitions violating the electric-dipole- radiation parity-change rule have been found near the strong allowed lines. The observations of Jacquinot [18] indicate that the forbidden lines are usually less than one-thousandth the strength of the corresponding allowed lines. If the need occurs, their wavelengths can be computed from the corresponding energy level differences.

Normally unobserved forbidden transitions in helium appear quite readily when the radiating gas is in a strong electric field. Investigations of the Stark effect in He I probably culminated in the beautiful work of Foster [19, 20], whose papers the reader may consult for other references.

Most investigations of the Zeeman effect in He I carried out in the last 30 years have been concerned with forbidden lines. The work of J. Brochard and P. Jacquinot [21] is rather comprehensive. A noteworthy achievement is the determination of the *g_j_*-value for 2*s* ^3^S_1_ to an accuracy of 1 ppm. by Drake et al. [22] using microwave techniques.

The quantitative intensity measurements of Crosswhite and Dieke [23] show striking changes in the relative intensities of various helium lines when only the gas pressure is changed. As in the vacuum ultraviolet region, the intensities given for lines above 2615 A are not valid for all commonly occurring excitation conditions.

C. J. Humphreys [24] has very kindly furnished unpublished quantitative intensity measurements for the infrared region beyond 10912 A. His source was an electrodeless tube filled to 3.5 mm (Hg), and care was taken to obtain precise intensities. Humphreys states that the intensity of the triplet at 10830 A is 5 to 10 times that of the singlet line at 20581 A which has intensity 20850 on his scale. Since this intensity ratio would not be much different at the pressure (7.5 mm) at which Crosswhite and Dieke [23] obtained an intensity of 105,000 for the 10830 triplet, it seems that these observers have used very nearly the same scale.

It is hoped that the intensities given in table 1 for lines from 2644 to 21132 A are on this reasonably consistent scale with the relative intensities of the stronger lines being useful for most sources operating at a few millimeters (Hg) pressure of helium. (The intensities of the weaker lines are especially dependent on excitation conditions other than pressure.) In addition to the article of Crosswhite and Dieke, almost every paper referred to here which gives intensities in the region 2644 to 10912 A has been consulted. The intensities in this region are also based in part on observations made at NBS.

## 3. Energy Levels

A new square array has been made for the ^4^He I spectrum. The first step in obtaining revised energy level values (based on the value 0.00 for the 1*s*^2 1^S_0_ ground state) was to fix the $2p^{3}P_{1}^{{}^{\circ}}—2p\;{\;}^{1}P_{1}^{{}^{\circ}}$ and $2p^{1}P_{1}^{{}^{\circ}}—3p\;{\;}^{1}P_{1}^{{}^{\circ}}$ separations. Herzberg [4] observed the vacuum ultraviolet transitions from each of these three odd levels to the ground state with an accuracy of ±0.0005 A at 500 to 600 A. From the best values for these separations as determined by the relevant observations in the air region, and from Herzberg’s wavenumbers for the vacuum ultraviolet lines, the positions of $2p\;{\;}^{3}P_{1}^{{}^{\circ}}$, $2p\;{\;}^{1}P_{1}^{{}^{\circ}}$, and $3p\;{\;}^{1}P_{1}^{{}^{\circ}}$ were fixed as given in table 2. With the ground state thus determined relative to the other energy levels, new values for all levels have been calculated from the best available measurements. The relative positions of the singlet and triplet systems are based primarily on Herzberg’s measurements of the intercombination lines $2p^{1}P_{1}^{{}^{\circ}}—3d\;{\;}^{3}D_{2}$ (6679.683 A) and $2p^{3}P_{1}^{{}^{\circ}}—3d\;{\;}^{1}D_{2}$ (5874.463 A). The resulting positions give good agreement with Mlle. Pilon’s measurement [25] of the 3*d* ^3^D—3*d* ^1^D separation, and it is unlikely that the intersystem connection is in error by more than 0.02 cm^−1^. A recent unpublished interferometric measment of $2s^{1}S_{0}—2p\;{\;}^{1}P_{1}^{{}^{\circ}}$ (4857.454 cm^−1^) has been combined with the average of two recent interferometric determinations of $2s{\;}^{1}S^{{}^{\circ}}—3p\;{\;}^{1}P_{1}^{{}^{\circ}}$ to obtain the $2p^{1}P_{1}^{{}^{\circ}}—3p\;{\;}^{1}P_{1}^{{}^{\circ}}$ separation. Since these measurements (the sources of which are given in table 1) are much more accurate than any other pair determining this separation, its value is based entirely on them. The positions of the higher terms relative to the ground state have been taken so that subtraction of the resulting level values for $2p\;{\;}^{3}P_{1}^{{}^{\circ}}$, $2p\;{\;}^{1}P_{1}^{{}^{\circ}}$, and $3p\;{\;}^{1}P_{1}^{{}^{\circ}}$ from the corresponding “observed” values of Herzberg yields, respectively, −0.11, +0.04, and +0.11 cm^−1^. Herzberg’s estimated uncertainty in the wavenumbers of the observed lines is +0.15 cm^−1^.

The measurements used here to determine the resolved fine structure intervals deserve some discussion, since all of these have been improved in the last few years. The $2p{\;}^{3}P_{1}^{{}^{\circ}}—2p\;{\;}^{3}P_{0}^{{}^{\circ}}$ splitting is taken from the paper of Brochard et al. [26], while the much smaller $2p{\;}^{3}P_{2}^{{}^{\circ}}—2p\;{\;}^{3}P_{1}^{{}^{\circ}}$ interval is a conversion to wavenumbers of the microwave frequency measurements by Wieder and Lamb [27]. (This last measurement gave the 2−1 splitting to a higher accuracy than is shown in the present table—for those who may need this accuracy the result of Wieder and Lamb is $2p{\;}^{3}P_{2}^{{}^{\circ}}—2p\;{\;}^{3}P_{1}^{{}^{\circ}}=2291.72\pm 0.36$ Mc/sec.) In 1929 Houston [28] gave the 3*p* ^3^P° intervals as 2—1 = 0.02 cm^−1^ and 1—0 = 0.27 cm^−1^. He had not resolved the smaller separation, but guessed at it from the analogy with the 2*p* ^3^P° term and his measurement of the $3p{\;}^{3}P_{2,1}^{{}^{\circ}}—{\;}^{3}P_{0}^{{}^{\circ}}$ splitting. At the time the first volume of AEL [5] appeared, it was thought that Houston’s measurements had been superseded by those of Gibbs and Kruger [29]. These latter observers found the $3p^{3}P_{2}^{{}^{\circ}}—3p\;{\;}^{3}P_{1}^{{}^{\circ}}$ and $3p^{3}P_{1}^{{}^{\circ}}—3p\;{\;}^{3}P_{0}^{{}^{\circ}}$ intervals to be 0.165 and 0.192 cm^−1^, respectively. These results proved in disagreement with theoretical expectations [30], which confirmed Houston’s assumption that the ratio of $np{\;}^{3}P_{2}^{{}^{\circ}}—np\;{\;}^{3}P_{1}^{{}^{\circ}}$ to $np{\;}^{3}P_{1}^{{}^{\circ}}—np\;{\;}^{3}P_{0}^{{}^{\circ}}$ should be roughly independent of *n.* However, only in 1951 did Fred et al. [31] point out that the intervals of Gibbs and Kruger were spurious. The error was apparently caused by self-reversal in the strong unresolved line at 3888 A, which arises from the $2s{\;}^{3}S_{1}—3p\;{\;}^{3}P_{2,1}^{{}^{\circ}}$ transitions. Bradley and Kuhn [32] and Brochard et al. [33] also obtained results in essential agreement with the early work of Houston. Brochard et al. [33] first resolved the $3p\;{\;}^{3}P_{2}^{{}^{\circ}}—3p\;{\;}^{3}P_{1}^{{}^{\circ}}$ splitting, and in 1957 they improved their accuracy [26]. The results of their optical measurements are in excellent agreement with the 3*p* ^3^P° intervals given in table 2, which are taken from the very accurate microwave observations by Wieder and Lamb [27]. The 2*p* and 3*p* triplet levels are given to four decimal places in table 2 in order to exhibit the accurately known term intervals. The inter-term separations are, of course, not known to this accuracy.

### Note added in proof

The results of very recent high-accuracy measurements of the 2*p* ^3^P° intervals in He I are given in reference [38].

Theoretical calculations of *np* ^3^P° fine structures in He I have been made by Breit [34] and Araki [30]. A more approximate, but simpler, treatment given in the article of Bethe and Salpeter [35] predicts that the splitting is independent of *n* except for an overall multiplying factor of *n*^−3^. This result holds for any series of terms in a helium-like atom. How well this approximation is obeyed in the 2*p* and 3*p* triplets of ^4^He I may be seen from table 3. The fine structure measurements by Brochard et al. [26] yield the results shown in table 4 for the 3*d* ^3^D and 4*d* ^3^D terms. The quite small ^3^D_3_—^3^D_2_ intervals (0.0030± 0.0008 cm^−1^ for 3*d*, and 0.0018±0.001 for 4*d* )are not accurately enough measured to check on the constancy of the interval ratio given in the table, and the values given can only be taken as indicative. Brochard et al. [26] compare their observations of all the known ^4^He I fine-structure intervals with the results of various theoretical calculations

The new positions given in table 2 for the 4*f* ^1^F° and 4*f* ^3^F° terms are based primarily on Mlle. Pilon’s measurements [25] of the 4*d* ^1^D—4*f* ^1^F° and 4*d* ^3^D— 4*f* ^3^F° separations. To a lesser extent they are also based on the measurements by C. J. Humphreys and H. J. Kostkowski [36] of the infrared transitions from the 4*f* terms to 3*d* terms. The agreement is within the expected error of Humphreys and Kostkowski, and the 5*f* terms positions given here are based entirely on their measurements.

In his investigations of the Stark effect in He I, J. S. Foster [19] observed transitions to the 2*p* ^1^P° level from terms of every possible *L*-value belonging to the configurations 1*s* 5*l*, 6*l*, and 7*l.* Since his measurements were made in rather strong electric fields, they cannot be used directly to obtain unperturbed energy levels. From a comparison of Foster’s estimated wavelengths for certain transitions at zero field with those calculated from the 6*f* and 7*f* term values in table 2, it appears that his wavelengths are probably systematically slightly in error. However, approximate term values have been obtained from Foster’s work by using his measured separations of the lines in a group $2p{\;}^{1}P_{1}^{{}^{\circ}}—nl^{3,1}$ *L* from one of these lines whose zero-field wavenumber was known. These separations could be corrected to zero-field strength by comparing Foster’s *nd* ^1^D—^1^P° separation with the known zero-field separation of these levels for each *n.* The term positions were also based on values found from plotting the very regular Rydberg denominators, particularly in the case of the 7*l* terms. In this manner the positions of terms belonging to the *5g*, 6*g*, 6*h, 7g*, and 7*h* configurations have been calculated and included in table 2.

New level values based on recent improved series measurements and the accurate connection between the singlet and triplet systems have obviated several features of the He-atom energy level scheme which were previously disturbing. The best previous list of helium energy levels (AEL [5], vol. 1), following Paschen-Götze, had the *nf* singlet series members falling below the corresponding triplets from *n* = 5 on. (Indeed, Humphreys and Kostkowski [36] listed both 4*f* ^1^F° and 5*f* ^1^F° slightly below the corresponding ^3^F° terms on the basis of their improved measurements in the infrared—however, they also assumed the old Paschen-Götze singlet-triplet connection based on series limit determinations.) Similarly, the earlier data indicated that the *nd* ^1^D series crossed over and fell below the *nd* ^3^D series at *n* = 9. It is now clear that this puzzling behavior was not real. Shenstone’s much improved measurements [17] of the *nd* triplet and singlet series from 8*d* on showed that the apparent crossing-over of those series at 9*d* was due to inaccurate observations. His measurements were essentially confirmed and the singlet series extended to 18*d* by Herzberg [4]. The new connection between the singlet and triplet systems, which raises the singlet system about 0.10 cm^−1^ relative to the triplets, has removed the slight crossing- over at 13*d* which remained after Shenstone’s work. This accurate connection is also mainly responsible for rendering the *nf* series quite regular with the singlets probably slightly above the triplets. The observations are, however, not sufficiently accurate above 4*f* to fix the relative positions of the terms. One can say, however, that beyond 5*f* the ^1^F° terms are probably not separated from the corresponding ^3^F° terms by more than 0.1 cm^−1^.

The energies for members of the series *np* ^1^P° from 11*p* on are taken from a series formula. Paschen and Götze apparently thought this method would give better values than any measurements available to them; since there have been no new observations, the series has been recalculated to 20*p.* Higher members of the ^3^P° series were obtained by converting wavelengths from Paschen-Götze to vacuum wavenumbers according to Edlén’s tables. Several numerical errors occurring in Paschen-Götze have been corrected.

Combining the limits obtained by Herzberg [4] for three separate series with energies for the three appropriate fixed terms from the new array yields the following values for the ionization energy of helium:

From the series 3*d* ^3^D—*nf* ^3^F°:

$$\begin{array}{c} 3d{\;}^{3}D+\text{limit}=186101.65\;{\text{cm}}^{-1}+12209.20\;{\text{cm}}^{-1}=\\ 198310.85\;{\text{cm}}^{-1}; \end{array}$$

from the series 2*p* ^3^p°_2,1_*—nd* ^3^D:

$$\begin{array}{c} 2p{\;}^{3}{P^{{}^{\circ}}}_{2,1}+\text{limit}=169086.89\;{\text{cm}}^{-1}+29223.90\;{\text{cm}}^{-1}=\\ 198310.79\;{\text{cm}}^{-1}; \end{array}$$

from the series 2*p* ^1^P°_1_—*nd* ^1^D_2_:

$$\begin{array}{c} 2p{\;}^{1}{P^{{}^{\circ}}}_{1}+\text{limit}=171135.00\;{\text{cm}}^{-1}+27175.78\;{\text{cm}}^{-1}=\\ 198310.78\;\text{cm}.^{-1}. \end{array}$$

The average of these values, 198310.81 cm^−1^, is in excellent agreement with the experimental ionization energy of ^4^He adopted by Herzberg in his paper, 198310.82±0.15 cm^−1^. The spread in the three values obtained here is reduced from that obtained by Herzberg, no doubt because the available data have already been averaged once in obtaining values for the fixed term of each series.

In conclusion, it might be mentioned that the excellent article by Bethe and Salpeter [35] on the theory of one- and two-electron atoms gives numerous references to theoretical work on helium-like atoms through 1956. The informative paper by Bengt Edlén [37] on experimental results for the He I isoelectro nic sequence will also prove helpful to those interested in two-electron atoms.

## Figures and Tables

**Table 1:** Wavelengths and classifications for the lines of the *^4^He I* spectrum The meanings of the symbols in the first two columns will be understood from the discussion in the text and the explanations given at the end of this table.

Observer		λ (vac)	Intensity	cm^−1^	Classification
1	2				

Kg	Kg	320.392	10	312118.	1*s*2*p*^3^P° — 2*p*^23^P
Su	CS	505.5001	2	197823.91	1*s*^2 1^S_0_ — 15*p* ${\;}^{1}P_{1}^{{}^{\circ}}$
Su	CS	505.6840	3	197751.94	1*s*^2 1^S_0_ —14*p* ${\;}^{1}P_{1}^{{}^{\circ}}$
Su	CS	505.9122	4	197662.75	1*s*^2 1^S_0_ 13*p* ${\;}^{1}P_{1}^{{}^{\circ}}$
Su	CS	506.2000	5	197550.36	1*s*^2 1^S_0_ —12*p* ${\;}^{1}P_{1}^{{}^{\circ}}$
Hp	CS	506.5702	7	197405.99	1*s*^2 1^S_0_ —11*p* ${\;}^{1}P_{1}^{{}^{\circ}}$
Hp	C	507.0576	10	197216.24	1*s*^2 1^S_0_ — 10*p* ${\;}^{1}P_{1}^{{}^{\circ}}$
Hp	C	507.7178	15	196959.79	1*s*^2 1^S_0_ — 9*p* ${\;}^{1}P_{1}^{{}^{\circ}}$
Hp	C	508.6431	20	196601.51	1*s*^2 1^S_0_ — 8*p* ${\;}^{1}P_{1}^{{}^{\circ}}$
Hp	C	509.9979	25	196079.24	1*s*^2 1^S_0_ — 7*p* ${\;}^{1}P_{1}^{{}^{\circ}}$
Su	C	510.2586	……….	195979.04	1*s*^2 1^S_0_ — 7*s* ^1^S_0_
Hp	C	512.0982	35	195275.04	1*s*^2 1^S_0_ — 6*p* ${\;}^{1}P_{1}^{{}^{\circ}}$
Su	C	512.5183	……….	195115.00	1*s*^2 1^S_0_ — 6*s* ^1^S_0_
Hp	C	515.6165	50	193942.57	1*s*^2 1^S_0_ — 5*p* ${\;}^{1}P_{1}^{{}^{\circ}}$
Su	C	516.3592	……….	193663.63	1*s*^2 1^S_0_ — 5*s* ^1^S_0_
Hp	C	522.2128	80	191492.82	1*s*^2 1^S_0_ — 4*p* ${\;}^{1}P_{1}^{{}^{\circ}}$
Su	C	523.7238	……….	190940.33	1*s*^2 1^S_0_ — 4*s* ^1^S_0_
Hz	C	537.0296	200	186209.47	1*s*^2 1^S_0_ — 3*p* ${\;}^{1}P_{1}^{{}^{\circ}}$
Su	C	537.3309	……….	186105.06	1*s*^2 1^S_0_ — 3*d* ^1^D_2_
Su	C	538.8956	……….	185564.68	1*s*^2 1^S_0_ — 3*p* ${\;}^{3}P_{1}^{{}^{\circ}}$
Su	C	540.9354	……….	184864.94	1*s*^2 1^S_0_ — 3*s* ^1^S_0_
Hz	C	584.3340	500	171135.00	1*s*^2 1^S_0_ — 2*p* ${\;}^{1}P_{1}^{{}^{\circ}}$
Hz	C	591.4117	20	169086.94	1*s*^2 1^S_0_ — 2*p* ${\;}^{3}P_{1}^{{}^{\circ}}$
Kg	C	601.4041	……….	166277.55	1*s*^2 1^S_0_ — 2*s* ^1^S_0_
		λ (air)			
	P–G	2615.184	……….	38226.82	2*s* ^3^S_1_ —22*p* ^3^P°
	P–G	2616.711	……….	38204.51	2*s* ^3^S_1_ — 21*p* ^3^P°
	P–G	2618.478	……….	38178.73	2*s* ^3^S_1_ —20*p* ^3^P°
	P–G	2620.534	……….	38148.78	2*s* ^3^S_1_ — 19*p* ^3^P°
	P–G	2622.947	……….	38113.68	2*s* ^3^S_1_ —18*p* ^3^P°
	P–G	2625.806	……….	38072.19	2*s* ^3^S_1_ —17*p* ^3^P°
	P–G	2629.229	……….	38022.62	2*s* ^3^S_1_ —16*p* ^3^P°
	P–G	2633.375	……….	37962.76	2*s* ^3^S_1_ —15*p* ^3^P°
	P–G	2638.462	……….	37889.58	2*s* ^3^S_1_ — 14*p* ^3^P°
	P–G	2644.802	2	37798.76	2*s* ^3^S_1_ — 13*p* ^3^P°
	P–G	2652.848	3	37684.12	2*s* ^3^S_1_ — 12*p* ^3^P°
	P–G	2663.271	4	37536.65	2*s* ^3^S_1_ —11*p* ^3^P°
	P–G	2677.135	5	37342.27	2*s* ^3^S_1_ — 10*p* ^3^P°
	P–G	2696.119	7	37079.35	2*s* ^3^S_1_ — 9*p* ^3^P°
	P–G	2723.191	10	36710.75	2*s* ^3^S_1_ — 8*p* ^3^P°
P–G	C	2763.804	20	36171.33	2*s* ^3^S_1_ — 7*p* ^3^P°
P–G	C	2829.076	40	35336.84	2*s* ^3^S_1_ — *6p* ^3^P°
	Me	2945.106	100	33944.71	2*s* ^3^S_1_ — *5p* ^3^P°
P–G	CS	3147.779	……….	31759.24	2*s* ^1^S_0_ —20*p* ${\;}^{1}P_{1}^{{}^{\circ}}$
P–G	CS	3150.713	……….	31729.66	2*s* ^1^S_0_ — 19*p* ${\;}^{1}P_{1}^{{}^{\circ}}$
P–G	CS	3154.156	……….	31695.03	2*s* ^1^S_0_ — 18*p* ${\;}^{1}P_{1}^{{}^{\circ}}$
P–G	CS	3158.234	……….	31654.10	2*s* ^1^S_0_ — 17*p* ${\;}^{1}P_{1}^{{}^{\circ}}$
P–G	CS	3163.114	……….	31605.27	2*s* ^1^S_0_ —16*p* ${\;}^{1}P_{1}^{{}^{\circ}}$
P–G	CS	3169.021	……….	31546.36	2*s* ^1^S_0_ —15*p* ${\;}^{1}P_{1}^{{}^{\circ}}$
P–G	CS	3176.267	……….	31474.39	2*s* ^1^S_0_ —14p ${\;}^{1}P_{1}^{{}^{\circ}}$
P–G?	CS	3185.293	……….	31385.20	2*s* ^1^S_0_ — 13*p* ${\;}^{1}P_{1}^{{}^{\circ}}$
	Me	3187.745	200	31361.07	2*s* ^3^S_1_ — 4*p* ^3^P°
P–G	CS	3196.742	2	31272.81	2*s* ^1^S_0_ — 12*p* ${\;}^{1}P_{1}^{{}^{\circ}}$
P–G	CS	3211.568	2	31128.44	2*s* ^1^S_0_ — 11*p* ${\;}^{1}P_{1}^{{}^{\circ}}$
	P–G	3231.266	3	30938.69	2*s* ^1^S_0_ — 10*p* ${\;}^{1}P_{1}^{{}^{\circ}}$
	P–G	3258.275	5	30682.24	2*s* ^1^S_0_ — 9p ${\;}^{1}P_{1}^{{}^{\circ}}$
P–G	C	3296.773	7	30323.96	2*s* ^1^S_0_ — 8*p* ${\;}^{1}P_{1}^{{}^{\circ}}$
P–G	C	3354.550	10	29801.69	2*s* ^1^S_0_ — 7*p* ${\;}^{1}P_{1}^{{}^{\circ}}$
P–G	C	3447.586	15	28997.49	2*s* ^1^S_0_ — 6p ${\;}^{1}P_{1}^{{}^{\circ}}$
	P–G	3450.22	……….	28975.4	2*p* ${\;}^{3}P_{2,1}^{{}^{\circ}}$—21 *d* ^3^D
	P–G	3453.21	……….	28950.3	2*p* ${\;}^{3}P_{2,1}^{{}^{\circ}}$—20*d* ^3^D
	Hz	3456.841	……….	28919.86	2*p* ${\;}^{3}P_{2,1}^{{}^{\circ}}$—19*d* ^3^D
	Hz	3461.000	……….	28885.11	2*p* ${\;}^{3}P_{2,1}^{{}^{\circ}}$—18*d* ^3^D
	Hz*	3465.925	……….	28844.07	2*p* ${\;}^{3}P_{2,1}^{{}^{\circ}}$—17*d* ^3^D
	Sh	3467.539	……….	28830.64	2*p* ${\;}^{3}P_{2,1}^{{}^{\circ}}$—17*s* ^3^S_1_
Hz	C	3471.818	……….	28795.11	2*p* ${\;}^{3}P_{2,1}^{{}^{\circ}}$—16*d* ^3^D
Hz	C	3471.943	……….	28794.07	2*p* ${\;}^{3}P_{0}^{{}^{\circ}}$ — 16*d* ^3^D_1_
	Sh	3473.764	……….	28778.98	2*p* ${\;}^{3}P_{2,1}^{{}^{\circ}}$—16*s* ^3^S_1_
Hz	C	3478.957	2	28736.02	2*p* ${\;}^{3}P_{2,1}^{{}^{\circ}}$—15*d* ^3^D
Hz	C	3479.083	……….	28734.98	2*p* ${\;}^{3}P_{0}^{{}^{\circ}}$ — 15*d* ^3^D_1_
	Sh	3481.355	……….	28716.23	2*p* ${\;}^{3}P_{2,1}^{{}^{\circ}}$—15*s* ^3^S_1_
Hz	C	3487.723	2	28663.80	2*p* ${\;}^{3}P_{2,1}^{{}^{\circ}}$—14*d* ^3^D
Hz	C	3487.850	……….	28662.76	2*p* ${\;}^{3}P_{0}^{{}^{\circ}}$ —14*d* ^3^D_1_
	Sh	3490.685	……….	28639.48	2*p* ${\;}^{3}P_{2,1}^{{}^{\circ}}$—14*s* ^3^S_1_
Hz	C	3498.645	3	28574.32	2*p* ${\;}^{3}P_{2,1}^{{}^{\circ}}$—13*d* ^3^D
Hz	C	3498.772	……….	28573.28	2*p* ${\;}^{3}P_{0}^{{}^{\circ}}$ —13*d* ^3^D_1_
	Sh	3502.379	2	28543.86	2*p* ${\;}^{3}P_{2,1}^{{}^{\circ}}$—13*s* ^3^S_1_
Hz	C	3512.512	4	28461.52	2*p* ${\;}^{3}P_{2,1}^{{}^{\circ}}$—12*d* ^3^D
Hz	C	3512.640	……….	28460.48	2*p* ${\;}^{3}P_{0}^{{}^{\circ}}$ —12*d* ^3^D_1_
	Sh	3517.317	2	28422.63	2*p* ${\;}^{3}P_{2,1}^{{}^{\circ}}$— 12*s* ^3^S_1_
Hz	C	3530.491	5	28316.58	2*p* ${\;}^{3}P_{2,1}^{{}^{\circ}}$—11*d* ^3^D
Hz	C	3530.621	……….	28315.54	2*p* ${\;}^{3}P_{0}^{{}^{\circ}}$ —11*d* ^3^D_1_
	Sh	3536.809	3	28266.00	2*p* ${\;}^{3}P_{2,1}^{{}^{\circ}}$—11*s* ^3^S_1_
	Hz	3554.415	7	28125.99	2*p* ${\;}^{3}P_{2,1}^{{}^{\circ}}$—10*d* ^3^D
Hz	C	3554.547	……….	28124.95	2*p* ${\;}^{3}P_{0}^{{}^{\circ}}$ — 10*d* ^3^D_1_
	Sh	3562.979	4	28058.39	2*p* ${\;}^{3}P_{2,1}^{{}^{\circ}}$—10*s* ^3^S_1_
	Hz	3587.270	10	27868.39	2*p* ${\;}^{3}P_{2,1}^{{}^{\circ}}$— 9*d* ^3^D
Hz	C	3587.405	2	27867.35	2*p* ${\;}^{3}P_{0}^{{}^{\circ}}$ — 9*d* ^3^D_1_
	Sh	3599.314	5	27775.15	2*p* ${\;}^{3}P_{2,1}^{{}^{\circ}}$— 9*s* ^3^S_1_
P–G	C	3599.448	2	27774.11	2*p* ${\;}^{3}P_{0}^{{}^{\circ}}$ — 9*s* ^3^S_1_
	Me	3613.643	30	27665.02	2*s* ^1^S_0_ — 5*p* ${\;}^{1}P_{1}^{{}^{\circ}}$
P–G	C	3634.232	15	27508.29	2*p* ${\;}^{3}P_{2,1}^{{}^{\circ}}$— 8*d* ^3^D
P–G	C	3634.369	2	27507.25	2*p* ${\;}^{3}P_{0}^{{}^{\circ}}$ — 8*d* ^3^D_1_
	Sh	3651.990	7	27374.53	2*p* ${\;}^{3}P_{2,1}^{{}^{\circ}}$ — 8*s* ^3^S_1_
P–G	C	3652.130	2	27373.49	2*p* ${\;}^{3}P_{0}^{{}^{\circ}}$ — 8*s* ^3^S_1_
	Me	3705.005	30	26982.84	2*p* ${\;}^{3}P_{2,1}^{{}^{\circ}}$— 7*d* ^3^D
P–G	C	3705.148	3	26981.80	2*p* ${\;}^{3}P_{0}^{{}^{\circ}}$ — 7*d* ^3^D_1_
	Hz	3725.130	……….	26837.07	2*p* ${\;}^{1}P_{1}^{{}^{\circ}}$ —18*d* ^1^D_2_
	Hz	3730.839	……….	26796.00	2*p* ${\;}^{1}P_{1}^{{}^{\circ}}$ —17*d* ^1^D_2_
P–G	C	3732.865	10	26781.46	2*p* ${\;}^{3}P_{2,1}^{{}^{\circ}}$— 7*s* ^3^S_1_
P–G	C	3733.010	3	26780.42	2*p* ${\;}^{3}P_{0}^{{}^{\circ}}$ — 7*s* ^3^S_1_
	Hz	3737.674	……….	26747.01	2*p* ${\;}^{1}P_{1}^{{}^{\circ}}$ —16*d* ^1^D_2_
	Hz, Sh	3745.943	……….	26687.96	2*p* ${\;}^{1}P_{1}^{{}^{\circ}}$ —15*d* ^1^D_2_
	Sh	3747.208	……….	26678.95	2*p* ${\;}^{1}P_{1}^{{}^{\circ}}$ — 15*s* ^1^S_0_
	Hz, Sh	3756.107	……….	26615.75	2*p* ${\;}^{1}P_{1}^{{}^{\circ}}$ —14*d* ^1^D_2_
	Sh	3757.670	……….	26604.67	2*p* ${\;}^{1}P_{1}^{{}^{\circ}}$ —14*s* ^1^S_0_
	Hz, Sh	3768.784	2	26526.22	2*p* ${\;}^{1}P_{1}^{{}^{\circ}}$ — 13*d* ^1^D_2_
	Sh	3770.751	……….	26512.38	2*p* ${\;}^{1}P_{1}^{{}^{\circ}}$ — 13*s* ^1^S_0_
	Hz, Sh	3784.862	2	26413.54	2*p* ${\;}^{1}P_{1}^{{}^{\circ}}$ — 12*d* ^1^D_2_
	Sh	3787.424	……….	26395.68	2*p* ${\;}^{1}P_{1}^{{}^{\circ}}$ —12*s* ^1^S_0_
	Hz, Sh	3805.740	3	26268.64	2*p* ${\;}^{1}P_{1}^{{}^{\circ}}$ — 11*d* ^1^D_2_
	CS	3809.105	……….	26245.44	2*p* ${\;}^{1}P_{1}^{{}^{\circ}}$ — 11*s* ^1^S_0_
	Mt	3819.6072	100	26173.275	2*p* ${\;}^{3}P_{2,1}^{{}^{\circ}}$ — 6*d* ^3^D
P–G	C	3819.758	10	26172.239	2*p* ${\;}^{3}P_{0}^{{}^{\circ}}$ — 6*d* ^3^D_1_
	Hz, Sh	3833.554	4	26078.06	2*p* ${\;}^{1}P_{1}^{{}^{\circ}}$ — 10*d* ^1^D_2_
	Sh	3838.100	2	26047.17	2*p* ${\;}^{1}P_{1}^{{}^{\circ}}$ — 10*s* ^1^S_0_
P–G	C	3867.475	30	25849.34	2*p* ${\;}^{3}P_{2,1}^{{}^{\circ}}$— 6*s* ^3^S_1_
P–G	C	3867.630	5	25848.30	2*p* ${\;}^{3}P_{0}^{{}^{\circ}}$ — 6*s* ^3^S_1_
	Hz	3871.791	5	25820.52	2*p* ${\;}^{1}P_{1}^{{}^{\circ}}$ — 9*d* ^1^D_2_
	Sh	3878.181	3	25777.98	2*p* ${\;}^{1}P_{1}^{{}^{\circ}}$ — 9*s* ^1^S_0_
	Me	3888.648	10000	25708.594	2*s* ^3^S_1_ — 3*p* ${\;}^{3}P_{2,1}^{{}^{\circ}}$
P–G	C	3926.534	7	25460.54	2*p* ${\;}^{1}P_{1}^{{}^{\circ}}$ — 8*d* ^1^D_2_
	Sh	3935.912	4	25399.88	2*p* ${\;}^{1}P_{1}^{{}^{\circ}}$ — 8*s* ^1^S_0_
	Mt	3964.7289	200	25215.271	2*s* ^1^S_0_ — 4*p* ${\;}^{1}P_{1}^{{}^{\circ}}$
P–G	C	4009.268	10	24935.16	2*p* ${\;}^{1}P_{1}^{{}^{\circ}}$ — 7*d* ^1^D_2_
	P–G	4023.973	5	24844.04	2*p* ${\;}^{1}P_{1}^{{}^{\circ}}$ — 7*s* ^1^S_0_
	Mt	4026.1912	500	24830.353	2*p* ${\;}^{3}P_{2,1}^{{}^{\circ}}$ — 5*d* ^3^D
P–G	C	4026.359	50	24829.317	2*p* ${\;}^{3}P_{0}^{{}^{\circ}}$ — 5*d* ^3^D_1_
	Mt	4120.815	120	24260.197	2*p* ${\;}^{3}P_{2,1}^{{}^{\circ}}$ — 5*s* ^3^S_1_
P–G	C	4120.992	15	24259.161	2*p* ${\;}^{3}P_{0}^{{}^{\circ}}$ — 5*s* ^3^S_1_
	Me	4143.761	30	24125.86	2*p* ${\;}^{1}P_{1}^{{}^{\circ}}$ — 6*d* ^1^D_2_
	Of	4168.967	5	23980.00	2*p* ${\;}^{1}P_{1}^{{}^{\circ}}$ — 6*s* ^1^S_0_
	Mt	4387.9294	100	22783.391	2*p* ${\;}^{1}P_{1}^{{}^{\circ}}$ — 5*d* ^1^D_2_
	Me	4437.551	30	22528.627	2*p* ${\;}^{1}P_{1}^{{}^{\circ}}$ — 5*s* ^1^S_0_
	Mt, Pé	4471.479	2000	22357.692	2*p* ${\;}^{3}P_{2,1}^{{}^{\circ}}$ — 4*d* ${\;}^{3}D_{3,2}^{{}^{\circ}}$
P–G	C	4471.682	250	22356.676	2*p* ${\;}^{3}P_{0}^{{}^{\circ}}$ — 4*d* ^3^D_1_
	Mt	4713.1455	300	21211.318	2*p* ${\;}^{3}P_{2,1}^{{}^{\circ}}$ — 4*s* ^3^S_1_
P–G	C	4713.376	40	21210.282	2*p* ${\;}^{3}P_{0}^{{}^{\circ}}$ — 4*s* ^3^S_1_
	Mt	4921.9310	200	20311.559	2*p* ${\;}^{1}P_{1}^{{}^{\circ}}$ — 4*d* ^1^D_2_
	Mt, Se-F	5015.6779	1000	19931.925	2*s* ^1^S_0_ — 3*p* ${\;}^{1}P_{1}^{{}^{\circ}}$
	Me	5047.738	100	19805.331	2*p* ${\;}^{1}P_{1}^{{}^{\circ}}$ — 4*s* ^1^S_0_
	Hz	5874.463	……….	17018.116	2*p* ${\;}^{3}P_{1}^{{}^{\circ}}$ — 3*d* ^1^D_2_
	Pé, Me	5875.621	15000	17014.760	2*p* ${\;}^{3}P_{2,1}^{{}^{\circ}}$— 3*d* ^3^D_3,2_
P–G	C	5875.966	2000	17013.763	2*p* ${\;}^{3}P_{0}^{{}^{\circ}}$ — 3*d* ^3^D_1_
	Pé, Me	6678.151	2000	14970.071	2*p* ${\;}^{1}P_{1}^{{}^{\circ}}$ — 3*d* ^1^D_2_
	Hz	6679.683	……….	14966.637	2*p* ${\;}^{1}P_{1}^{{}^{\circ}}$ — 3*d* ^3^D_2_
	Me	7065.190	5000	14150.000	2*p* ${\;}^{3}P_{2,1}^{{}^{\circ}}$— 3*s* ^3^S_1_
Of	C	7065.707	600	14148.964	2*p* ${\;}^{3}P_{0}^{{}^{\circ}}$ — 3*s* ^3^S_1_
	Me, Of	7281.349	1000	13729.936	2*p* ${\;}^{1}P_{1}^{{}^{\circ}}$ — 3*s* ^1^S_0_
P-Rt	C	7816.15	10	12790.51	3*s* ^3^S_1_ — 7*p* ^3^P°
Mg-D	C	8361.69	20	11956.02	3*s* ^3^S_1_ — 6*p* ^3^P°
Hz†	C	8444.48	……….	11838.81	*3p* ${\;}^{3}P_{2,1}^{{}^{\circ}}$—11*d* ^3^D
	Hz†	8518.04	……….	11736.57	3*s* ^1^S_0_ — 8*p* ${\;}^{1}P_{1}^{{}^{\circ}}$
	Hz	8528.991	……….	11721.495	3*d* ^3^D — 15*f* ^3^F°
	Hz†	8582.65	……….	11648.21	$\left\{ \begin{array}{l} 3p{\;}^{3}P^{{}^{\circ}}—10d{\;}^{3}D\\ 3d{\;}^{3}D—14f{\;}^{3}F^{{}^{\circ}} \end{array}\right.$
	Hz	8648.257	……….	11559.85	3*d* ^3^D — 13*f* ^3^F°
	Hz	8733.431	……….	11447.11	3*d* ^3^D — 12*f* ^3^F°
Hz†	C	8776.74	5	11390.62	3*p* ${\;}^{3}P_{2,1}^{{}^{\circ}}$— 9*d* ^3^D
	Hz	8845.373	2	11302.24	3*d* ^3^D — 11*f* ^3^F°
P-Rt	C	8914.74	5	11214.30	3*s* ^1^S_0_ — *7p* ${\;}^{1}P_{1}^{{}^{\circ}}$
	Hz	8996.978	5	11111.79	3*d* ^3^D —10*f* ^3^F°
Mg-D	C	9063.27	15	11030.52	3*p* ${\;}^{3}P_{2,1}^{{}^{\circ}}$— 8*d* ^3^D
Mg-D	C	9085.45	2	11003.59	*3p* ${\;}^{1}P_{1}^{{}^{\circ}}$ —10*d* ^1^D_2_
Mg-D	C	9174.52	5	10896.76	3*p* ${\;}^{3}P_{2,1}^{{}^{\circ}}$— 8*s* ^3^S_1_
	Hz	9210.337	20	10854.39	3*d* ^3^D — 9*f* ^3^F°
	Mg-D	9213.1	2	10851.1	3*d* ^1^D_2_ — 9*f* ${\;}^{1}F_{3}^{{}^{\circ}}$
Mg-D	C	9303.19	3	10746.05	3*p* ${\;}^{1}P_{1}^{{}^{\circ}}$ — 9*d* ^1^D_2_
P-Rt	C	9463.61	100	10563.89	3*s* ^3^S_1_ — 5*p* ^3^P°
P-Rt	C	9516.60	40	10505.07	3*p* ${\;}^{3}P_{2,1}^{{}^{\circ}}$ — 7*d* ^3^D
P-Rt	C	9516.87	5	10504.78	3*p* ${\;}^{3}P_{0}^{{}^{\circ}}$ — 7*d* ^3^D_1_
	Mg-D	9526.17	30	10494.52	3*d* ^3^D — 8*f* ^3^F°
	Mg-D	9529.27	10	10491.10	*3d* ^1^D_2_ — 8*f* ${\;}^{1}F_{3}^{{}^{\circ}}$
Mg-D	C	9552.89	3	10465.17	*3d* ^3^D — 8*p* ^3^P°
P-Rt	C	9603.42	10	10410.10	3*s* ^1^S_0_ — 6*p* ${\;}^{1}P_{1}^{{}^{\circ}}$
Mg-D	C	9625.64	5	10386.07	3*p* ${\;}^{1}P_{1}^{{}^{\circ}}$ — 8*d* ^1^D_2_
Mg-D	C	9682.19	2	10325.41	3*p* ${\;}^{1}P_{1}^{{}^{\circ}}$ — 8s ^1^S_0_
P-Rt	C	9702.60	30	10303.69	3*p* ${\;}^{3}P_{2,1}^{{}^{\circ}}$— 7*s* ^3^S_1_
	Mg-D	10027.73	60	9969.61	3*d* ^3^D — 7*f* ^3^F°
	Mg-D	10031.16	20	9966.20	*3d* ^3^D_1_ — 7*f* ${\;}^{1}F_{3}^{{}^{\circ}}$
Mg-D	C	10072.04	5	9925.75	3*d* ^3^D — 7*p* ^3^P°
Mg-D	C	10138.50	10	9860.69	3*p* ${\;}^{1}P_{1}^{{}^{\circ}}$ — 7*d* ^1^D_2_
Mg-D	C	10233.06	3	9769.57	3*p* ${\;}^{1}P_{1}^{{}^{\circ}}$ — 7*s* ^1^S_0_
Mg-D	C	10311.23	100	9695.504	3*p* ${\;}^{3}P_{2,1}^{{}^{\circ}}$ — 6*d* ^3^D
	C	10311.54	15	9695.220	3*p* ${\;}^{3}P_{0}^{{}^{\circ}}$ — 6*d* ^3^D_1_
Mg	C	10667.65	30	9371.57	3*p* ${\;}^{3}P_{2,1}^{{}^{\circ}}$ — 6*s* ^3^S_1_
	C	10667.98	……….	9371.28	3*p* ${\;}^{3}P_{0}^{{}^{\circ}}$ — 6*s* ^3^S_1_
Mg	C	10829.088	10000	9231.859	2*s* ^3^S_1_ — 2*p* ${\;}^{3}P_{0}^{{}^{\circ}}$
Mg	C	10830.248	30000	9230.871	2*s* ^3^S_1_ — 2*p* ${\;}^{3}P_{1}^{{}^{\circ}}$
Mg	C	10830.337	50000	9230.795	2*s* ^3^S_1_ — 2*p* ${\;}^{3}P_{2}^{{}^{\circ}}$
Mg	C	10902.16	……….	9169.98	*3d* ^1^D_2_ — 6*p* ${\;}^{1}P_{1}^{{}^{\circ}}$
	Mg	10912.92	120*	9160.94	*3d* ^3^D — 6*f* ^3^F°
	Mg	10916.98	34	9157.53	*3d* ^1^D_2_ — 6*f* ${\;}^{1}F_{3}^{{}^{\circ}}$
Mg	C	10996.56	……….	9091.26	3*d* ^3^D — 6*p* ^3^P°
Mg	C	11013.07	16	9077.63	3*s* ^1^S_0_ — 5*p* ${\;}^{1}P_{1}^{{}^{\circ}}$
Mg	C	11045.00	17	9051.39	3*p* ${\;}^{1}P_{1}^{{}^{\circ}}$ — 6*d* ^1^D_2_
Mg	C	11225.90	……….	8905.53	3*p* ${\;}^{1}P_{1}^{{}^{\circ}}$ — 6s ^1^S_0_
Mg	C	11969.07	440	8352.584	3*p* ${\;}^{3}P_{2,1}^{{}^{\circ}}$— 5*d* ^3^D
	C	11969.48	……….	8352.298	3*p* ${\;}^{3}P_{0}^{{}^{\circ}}$ — 5*d* ^3^D_1_
Mg	C	12527.51	190	7980.25	3*s* ^3^S_1_ — 4*p* ^3^P°
	C	12755.66	……….	7837.51	3*d* ^1^D_2_ — 5*p* ${\;}^{1}P_{1}^{{}^{\circ}}$
	Hu-Ko	12784.79	810	7819.66	3*d* ^3^D — 5*f* ^3^F°
	Hu-Ko	12790.27	250	7816.31	3*d* ^1^D_2_ — 5*f* ${\;}^{1}F_{3}^{{}^{\circ}}$
Hu-Pl	C	12845.95	61	7782.426	3*p* ${\;}^{3}P_{2,1}^{{}^{\circ}}$— 5*s* ^3^S_1_
	C	12846.42	……….	7782.142	3*p* ${\;}^{3}P_{0}^{{}^{\circ}}$ — 5s ^3^S_1_
Hu-Pl	C	12968.44	100	7708.920	3*p* ${\;}^{1}P_{1}^{{}^{\circ}}$ — 5*d* ^1^D_2_
	C	12984.93	……….	7699.13	3*d* ^3^D — 5*p* ^3^P°
Hu-Pl	C	15083.66	120	6627.881	3*s* ^1^S_0_ — 4*p* ${\;}^{1}P_{1}^{{}^{\circ}}$
Hu-Ko	C	17002.36	} 4600	5879.925	3*p* ${\;}^{3}P_{2,1}^{{}^{\circ}}$ — 4*d* ^3^D
	C	17003.15		5879.657	3*p* ${\;}^{3}P_{0}^{{}^{\circ}}$ — 4*d* ^3^D_1_
Hu-Pl	C	18555.55	13	5387.752	3*d* ^1^D_2_ — 4*p* ${\;}^{1}P_{1}^{{}^{\circ}}$
Hu-Ko	C	18685.96	7250	5350.15	3*d* ^3^D — 4*f* ^3^F°
Hu-Ko	C	18696.94	3175	5347.01	3*d* ^1^D_2_ *—* 4*f* ${\;}^{1}F_{3}^{{}^{\circ}}$
Hu-Pl	C	19089.37	1100	5237.088	3*p* ${\;}^{1}P_{1}^{{}^{\circ}}$ — 4*d* ^1^D_2_
Hu-Pl	C	19543.13	130	5115.49	3*d* ^3^D *—* 4*p* ^3^P°
	Eu	20581.30	20850	4857.454	2*s* ^1^S_0_ — 2*p* ${\;}^{1}P_{1}^{{}^{\circ}}$
Hu-Pl	C	21120.04	} 600	4733.547	3*p* ${\;}^{3}P_{2,1}^{{}^{\circ}}$ — 4*s* ^3^S_1_
	C	21121.31		4733.263	3*p* ${\;}^{3}P_{0}^{{}^{\circ}}$ — 4*s* ^3^S_1_
Hu-Pl	C	21132.04	95	4730.860	3*p* ${\;}^{1}P_{1}^{{}^{\circ}}$ — 4*s* ^1^S_0_

* From 10912 A on, the intensities are measurements by C. J. Humphreys [24].

Explanation of Symbols C—Wavelength calculated from vacuum wavenumber difference of relevant energy levels from table 2. CS—Same as C, the upper energy level being obtained from a series formula. B—Rs—J. C. Boyce and H. A. Robinson, J. Opt. Soc. Am. **26**, 133 (1936). Hp—J. J. Hopfield, Astrophys. J. **72**, 133 (1930). Hu—C. J. Humphreys, unpublished wavelength, privately communicated to author, August 1959. Hu—Ko—C. J. Humphreys and H. J. Kostkowski, J. Research NBS **49**, 73 (1952). Hu—Pl—C. J. Humphreys and E. Paul, Jr., NAVORD Report 4589, 25 (1956); J. Opt, Soc. Am. **46**, 999 (1956). Hz—G. Herzberg, Proc. Roy. Soc. (London) [A] **248**, 309 (1958). Hz*— The air wavelength given for this line by Herzberg (above) is incorrect; the wavelength in his paper is actually the vacuum wavelength. Hz†—Unpublished observations by Herzberg, privately communicated to the author. Of these lines, only 8776 A has been previously recorded (P-Rt); Herzberg’s observed value of 8776.725 ±0.02 A is in good agreement with the calculated wavelength. The value 8518.040±0.02 A from Herzberg figured in determining the position of $8p\;{\;}^{1}P_{1}^{{}^{\circ}}$, and his measurement of 8444 A is the same as the calculated value. Kg—P. G. Kruger, Phys. Rev. **36**, 855 (1930). Mt—W. C. Martin, J. Opt, Soc. Am. (to be published). The vacuum wavelengths given in table 2 of this reference have been converted to air values according to the formula of Edlén. These wavelengths were further decreased by 0.0002 A for inclusion here because such a correction to the values assumed by Martin for the mercury-198 standards at 5462 A and 4359 A appeared probable at the time the new array was worked out, It now appears the correction should at most have been —0.0001 A; except for 5015 A, either correction is insignificant compared to the probable errors. Me—P. W. Merrill, Bul. BS **14**, 159 (1917); Astrophys. J. **46**, 357 (1917). Merrill’s wavelengths have all been increased by 0.002 A because of a systematic difference found to exist throughout the visible range between his measurements and those of Martin (above). Mg—W. F. Meggers, J. Research NBS **14**, 487 (1935). Mg—D—W. F. Meggers and G. H. Dieke, BS J. Research **9**, 121 (1932). Of—H. C. Offerhaus, Physica **3**, 309 (1923). P—G—F. Paschen and R. Götze, Seriengesetze der Linien-spectren, p. 26 (Julius Springer, Berlin, 1922). P—Rt—F. Paschen and R. Ritschl, Ann. Physik **18**, 867 (1933). In table 8a of this paper the designations “3 ^3^P_2_” and “3 ^3^P_1_” should be replaced by “3 ^3^P_2,1_” and “3 ^3^P_0_”, respectively (see text). Pé—A. Pérard, Rev. Optique **7**, 1 (1928). Se—F—G. W. Series and J. C. Field, Proceedings of the Symposium on Interferometry, Teddington, England, June 1959 (to be published) Sh—A. G. Shenstone, unpublished measurements Su—T. Suga, Sci. Papers Inst. Phys. Chem. Research (Tokyo) **34**, 7 (1937).

(Where two or more symbols separated by commas are given in the second column, a weighted average has been taken.)

**Table 2:** Energy levels of *^4^He I*

Term designation	*J*	Level

1*s*^2 1^S	0	0.00 ±0.15
2*s* ^3^S	1	159856.069
2*s* ^1^S	0	166277.546
2*p* ^3^P°	$\{\;\begin{array}{c} 2\\ 1\\ 0 \end{array}$	*169086.8636* *169086.9400* *169087.9280*
2*p* ^1^p°	1	*171135.000*
3*s* ^3^S	1	183236.892
3*s* ^1^S	0	184864.936
3*p* ^3^P°	$\{\;\begin{array}{c} 2\\ 1\\ 0 \end{array}$	*185564.6540* *185564.6760* *185564.9466*
3*d* °*D*	$\{\;\begin{array}{c} 3\\ 2\\ 1 \end{array}$	186101.643 186101.646 186101.691
3*d* ^1^D	2	186105.065
3*p* ^1^P°	1	*186209.471*
4*s* ^3^S	1	190298.210
4*s* ^1^S	0	190940.331
4*p* ^3^P°	2, 1, 0	*191217.14*
4*d* ^3^D	$\{\;\begin{array}{c} 3\\ 2\\ 1 \end{array}$	191444.583 191444.585 191444.604
4*d* ^1^D	2	191446.559
4*f* ^3^F°	4, 3, 2	*191451.80*
4*f* ^1^F°	3	*191452.08*
4*p* ^1^P°	1	*191492.817*
5*s* ^3^S	1	193347.089
5*s* ^1^S	0	193663.627
*5p* ^3^P°	2, 1, 0	*193800.78*
5*d* ^3^D	3, 2, 1	193917.245
5*d* ^1^D	2	193918.391
5*f* ^3^F°	4, 3, 2	*193921.31*
5*f* ^1^F°	3	*193921.37*
5*g* ^3,1^G	5, 4, 4, 3	193922.5
5*p* ^1^P°	1	*193942.57*
6*s* ^3^S	1	194936.23
6*s* ^1^S	0	195115.00
6*p* ^3^P°	2, 1, 0	*195192.91*
6*d* ^3^D	3, 2, 1	195260.167
6*d* ^1^D	2	195260.86
6*f* ^3^F°	4, 3, 2	*195262.59*
6*f* ^1^F°	3	*195262.59*
6*g* ^3,1^G	5, 4, 4, 3	195263.2
6*h* ^3,1^H°	6, 5, 5, 4	*195263.8*
6*p* ^1^P°	1	*195275.04*
7*s* ^3^S	1	195868.35
7*s* ^1^S	0	195979.04
7*p* ^3^P°	2, 1, 0	*196027.40*
7*d* ^3^D	3, 2, 1	196069.73
7*d* ^1^D	2	196070.16
7*f* ^3^F°	4, 3, 2	*196071.26*
7*f* ^1^F°	3	*196071.26*
7*g* ^3,1^G	5, 4, 4, 3	196071.7
7*h* ^3,1^H°	6, 5, 5, 4	*196072.0*
7*i* ^3,1^I^a^	7, 6, 6, 5	……….
7*p* ^1^P°	1	*196079.24*
8*s* ^3^S	1	196461.42
8*s* ^1^S	0	196534.88
8*p* ^3^P°	2, 1, 0	*196566.82*
8*d* ^3^D	3, 2, 1	196595.18
8*d* ^1^D	2	196595.54
8*f* ^3^F°	4, 3, 2	*196596.17*
8*f* ^1^F°	3	*196596.16*
8*p* ^1^P°	1	*196601.51*
9*s* ^3^S	1	196862.04
9*s* ^1^S	0	196912.98
9*p* ^3^P°	2, 1, 0	*196935.42*
9*d* ^3^D	3, 2, 1	196955.28
9*d* ^1^D	2	196955.52
9*f* ^3^F°	4, 3, 2	*196956.04*
9*f* ^1^F°	3	*196956.2*
9*p* ^1^P°	1	*196959.79*
10*s* ^3^S	1	197145.28
10*s* ^1^S	0	197182.17
10*p* ^3^P°	2, 1, 0	*197198.34*
10*d* ^3^D	3, 2.1	197212.88
10*d* ^1^D	2	197213.06
10*f* ^3^F°	4, 3, 2	*197213.44*
10*p* ^1^P°	1	*197216.24*
11*s* ^3^S	1	197352.89
11*s* ^1^S	0	b197380.44
11*p* ^3^P°	2, 1, 0	*197392.72*
11*d* ^3^D	3, 2, 1	197403.47
11*d* ^1^D	2	197403.64
11*f* ^3^F°	4, 3, 2	*197403.89*
11*p* ^1^P°	1	c*197405.99*
12*s* ^3^S	1	197509.52
12*s* ^1^S	0	197530.68
12*p* ^3^P°	2, 1, 0	*197540.19*
12*d* ^3^D	3, 2, 1	197548.41
12*d* ^1^D	2	197548.54
12*f* ^3^F°	4, 3, 2	*197548.76*
12*p* ^1^P°	1	*197550.36*
13*s* ^3^S	1	197630.75
13*p* ^3^P°	2, 1, 0	*197654.83*
13*s* ^1^S	0	d197647.38
13*d* ^3^D	3, 2, 1	197661.21
13*d* ^1^D	2	197661.22
13*f* ^3^F°	4, 3, 2	*197661.50*
13*p* ^1^P°	1	*197662.75*
14*s* ^3^S	1	197726.37
14*s* ^1^S	0	197739.67
14*p* ^3^P°	2, 1, 0	*197745.65*
14*d* ^3^D	3, 2, 1	197750.69
14*d* ^1^D	2	197750.75
14*f* ^3^F°	4, 3, 2	……….
14*p* ^1^F°	1	*197751.94*
15*s* ^3^S	1	197803.12
15*s* ^1^S	0	197813.95
15*p* ^3^P°	2, 1, 0	e*197818.83*
15*d* ^3^D	3, 2, 1	197822.91
15*d* ^1^D	2	197822.96
15*f* ^3^F°	4, 3, 2	*197823.15*
15*p* ^1^P°	1	*197823.91*
16*s* ^3^S	1	197865.87
16*p* ^3^P°	2, 1, 0	*197878.69*
16*d* ^3^D	3, 2, 1	197882.00
16*d* ^1^D	2	197882.01
16*p* ^1^P°	1	*197882.82*
17*s* ^3^S	1	197917.53
17*p* ^3^P°	2, 1, 0	*197928.26*
17*d* ^3^D	3, 2, 1	197930.96
17*d* ^1^D	2	197931.00
17*p* ^1^P°	1	*197931.65*
18*p* ^3^P°	2, 1, 0	*197969.75*
18*d* ^3^D	3, 2, 1	197972.00
18*d* ^1^D	2	197972.07
18*p* ^1^P°	1	*197972.58*
19*p* ^3^P°	2, 1, 0	*198004.85*
19*d* ^3^D	3, 2, 1	198006.75
19*p* ^1^P°	1	*198007.21*
20*p* ^3^P°	2, 1, 0	*198034.80*
20*d* ^3^D	3, 2, 1	f198036.4
20*p* ^1^p°	1	*198036.79*
21*p* ^3^P°	2, 1, 0	*198060.58*
21*d* ^3^D	3, 2, 1	198062.3
22*p* ^3^P°	2, 1, 0	*198082.89*
He II(^2^S_0½_)	**Limit**	**198310.81**
2*p*^2 3^P	2, 1, 0	481205.

a Although it is not possible to give the unnerturbed position of these terms, Foster [19] found transitions from them to 2*p* ^1^P° in the spectrum emitted by helium atoms in a strong electric field.

b Level value found by plotting the Rydberg denominators of the *ns* ^1^S series.

c The *np* ^1^p° levels were obtained from a series formula from 11*p* on.

d An incorrect value was given for this term bv Paschen-Götze. The wavelength listed there for the transition $2p\;{\;}^{1}P_{1}^{{}^{\circ}}—13s\;{\;}^{1}S_{0}$ is in error.

e The value given for this term in Paschen-Götze is incorrect.

f The energy for this term was calculated from a series formula. The values of Paschen-Götze for 18*d*, 19*d*, ^3^D are in disagreement with those of Herzberg by about 0.5 cm^−1^.

**Table 3:** Fine-structure regularities observed fcr the 2p and 3p triplets in *He I*.

*np*	$\frac{{\;}^{3}P_{2}^{{}^{\circ}}—{\;}^{3}P_{0}^{{}^{\circ}}}{{\;}^{3}P_{2}^{{}^{\circ}}—{\;}^{3}P_{1}^{{}^{\circ}}}$	$n^{3}\left({\;}^{3}P_{2}^{{}^{\circ}}—{\;}^{3}P_{0}^{{}^{\circ}}\right)$

2*p*	13.9	8.523 cm^−1^
3*p*	13.3	7.90

**Table 4:** Observed fine-structure ratios and n^−3^ dependence for the 3d and I+d triplets of *He I*.

*nd*	$\frac{{\;}^{3}D_{3}—{\;}^{3}D_{1}}{{\;}^{3}D_{3}—{\;}^{3}D_{2}}$	*n*^3^(^3^D_3_—^3^D_1_)

3*d*	16	1.30 cm^−1^
4*d*	11	1.31

## Footnotes

1 Figures in brackets indicate the literature references3 on pages 22 and 23.

## References

[1] Moore CE, Multiplet A (1945). Table of Astrophysical Interest.

[2] Paschen F, Götze R (1922). Seriengesetze der Linienspektren.

[3] Kayser H, Konen H (1934). Handbuch der Spectroscopie.

[4] Herzberg G (1958). Proc Roy Soc (London) [A].

[5] Moore CE (1949). Atomic Energy Levels. NBS Circ.

[6] Wu T-Y (1944). Phys Rev.

[7] Edlén B (1934). Nova Acta Reg Soc Sci Uppsala [IV].

[8] Kruger PG (1930). Phys Rev.

[9] Holϕien E (1958). Proc Phys Soc (London).

[10] Compton KT, Boyce JC, Franklin J (1928). Inst.

[11] Lyman T (1924). Astrophys J.

[12] Suga T (1937). Sci Papers Inst Phys Chem Research (Tokyo).

[13] Bomke H (1935). Physik Z.

[14] Hopfield JJ (1930). Astrophys J.

[15] Paschen F (1929). Sitz Berlin Akad Wiss.

[16] Boyce JC, Robinson HA, Opt J (1936). Soc Am.

[b17-jresv64an1p19_a1b] 17A.G. Shenstone, unpublished measurements; a list of energy levels based on this work appears at the end of [5], vol. III.

[18] Jacquinot P (1939). Compt Rend.

[19] Foster JS (1924). Phys Rev.

[20] Foster JS (1927). Proc Roy Soc (London).

[21] Brochard J, Jaquinot P (1945). Ann Phys.

[22] Drake, Hughes, Lurio, White (1958). Phys Rev.

[23] Crosswhite HM, Dieke GH (1957). Am Inst Phys Handbook.

[b24-jresv64an1p19_a1b] 24C.J. Humphreys, private communication, June, 1959.

[25] Pilon A-M (1954). J phys radium.

[26] Brochard J, Chabbal R, Chantrel H, Jacquinot P (1957). J phys radium.

[27] Wieder I, Lamb WE (1957). Phys Rev.

[28] Houston WV (1927). Proc Nat Acad Sci US.

[29] Gibbs RC, Kruger PG (1931). Phys Rev.

[30] Araki G (1937). Proc Phys Math Soc Japan.

[31] Fred M, Tomkins FS, Brody JK, Hamermesh M (1951). Phys Rev.

[32] Bradley LC, Kuhn H (1951). Proc Roy Soc (London).

[33] Brochard J, Chabbal R, Chantrel H, Jacquinot P (1952). J phys radium.

[34] Breit G (1930). Phys Rev.

[35] Bethe HA, Salpeter EE (1957). Quantum Mechanics of One- and Two-Electron Atoms.

[36] Humphreys CJ, Kostkowski HJ, Research J (1952). NBS.

[37] Edlén B (1952). Arkiv Fysik.

[38] Colegrove FD, Franken PA, Lewis RR, Sands RH (1959). Phys Rev Letters.

